# A dose escalation trial for the combination of erlotinib and sirolimus for recurrent malignant gliomas

**DOI:** 10.1007/s11060-012-0960-y

**Published:** 2012-08-24

**Authors:** Phioanh Leia Nghiemphu, Albert Lai, Richard M. Green, David A. Reardon, Timothy Cloughesy

**Affiliations:** 1 Department of Neurology, University of California, 710 Westwood Plaza, Reed 1-230, Los Angeles, CA 90230 USA; 2 Department of Neurology, Kaiser Permanente, Los Angeles, CA USA; 3 Center for Neuro-Oncology, Dana-Farber Cancer Institute, Boston, MA USA

**Keywords:** Erlotinib, Sirolimus, Recurrent gliomas, Combination study

## Abstract

In order to achieve higher dosages than previously used in clinical trials, we conducted a phase I trial to determine the maximum tolerated dose (MTD) for the combination of erlotinib and sirolimus for the treatments of recurrent malignant gliomas. Patients with pathologically proven World Health Organization (WHO) grade III glioma and grade IV glioblastoma and radiographically proven tumor recurrence were eligible for this study. Treatments included once daily erlotinib, which was given alone for the first 7 days of treatments, then in combination with once daily sirolimus. Sirolimus was given with a loading dose on day 8 followed by a maintenance dose starting on day 9. Dose-limiting toxicity (DLT) was determined over the first 28 days of treatments, and the MTD was determined in a 3 + 3 classic study design. 19 patients were enrolled, and 13 patients were eligible for MTD determination. The MTD was determined to be 150 mg daily for erlotinib and 5 mg daily (after a 15 mg loading dose) for sirolimus. The DLTs included rash and mucositis (despite maximal medical managements), hypophosphatemia, altered mental status, and neutropenia. The combination of erlotinib and sirolimus is difficult to tolerate at dosages higher than previously reported in phase II trials.

## Introduction

The incidence of primary malignant brain and central nervous system tumors in the United States is about 22,000 per year. These tumors cause over 13,000 deaths per year [1]. Of all malignant primary brain and central nervous system tumors, gliomas are the most common, with more than half of the cases comprising of malignant gliomas (World Health Organization, WHO grade III and IV). Despite optimal treatment with surgery, radiation therapy and chemotherapy, the prognosis remains poor. In the most aggressive type, grade IV glioblastoma (GBM), almost 90 % of patients will have tumor progression by 2 years after the standard treatments of radiation therapy combined with temozolomide chemotherapy [2]. At recurrence, treatments with alkylating chemotherapies or biological agents result only in a 6 months progression free survival (PFS) of 28–31 % for patients with WHO grade III and 15–16 % for those with grade IV [3, 4], and slightly longer at 29–45 % for those with grade IV treated with bevacizumab [5, 6]. Thus, more effective treatments at recurrence still need to be defined.

Targeting molecular aberrant pathways have been one option to enhance treatments against malignant gliomas, including inhibition of the epidermal growth factor receptor (EGFR). EGFR is activated by the ligand EGF which in turn activates multiple cell signaling pathways and modulates tumor cell division, invasion and apoptosis [7]. In addition, EGFR activation also indirectly affects factors that play an important role in tumor cell survival and growth such as vascular endothelial growth factor directed angiogenesis [8]. Dysregulation of EGFR via overexpression, amplification, and mutation affects the majority of malignant gliomas [9–11]. Two different types of mutations to the *EGFR* gene in GBM have been discovered. One mutation with deletion of several exons in the gene, EGFRvIII, leads to constitutive activation of the receptor. Several other missense mutations in the extracellular domain also lead to increased activation of the receptor [12]. Recently, The Cancer Genome Atlas project confirmed that about 45 % of GBM tumors harbor either focal amplification, mutation, or both of the *EGFR* gene[13], confirming that EGFR is a critical mediator of GBM pathogenesis and therefore represents a potentially important therapeutic target.

There have been several trials of EGFR inhibitors in GBM, mainly using small molecular ATP-competitive tyrosine kinase inhibitors Several phase II trials of EGFR inhibitors such as erlotinib or gefitinib have demonstrated limited effectiveness of these agents for gliomas[14]. Looking at tumor response to erlotinib or gefitinib, Mellinghoff et al. [15] found that EGFRvIII sensitize tumors to EGFR kinase inhibitors, and loss of the phosphotase tensin homologue of ten (PTEN) tumor suppressor would impair the response to such inhibitors. In tumor tissues of subjects given erlotinib or gefitinib for the treatment of recurrent GBM, EGFRvIII/PTEN protein coexpression was significantly associated with clinical response. The constitutively active mutant EGFRvIII is known to strongly and persistently activate the phosphatidylinositol 3′ kinase (PI3K) signaling pathway, which provides critical information for cell survival, proliferation, and motility. The loss of PTEN, which can be seen in 36 % of glioblastoma [13], may promote cellular resistance to EGFR kinase inhibitor therapy by dissociating EGFR inhibition from downstream PI3K pathway inhibition. Treatments targeting the PI3K pathway using mTOR-C1 inhibitors, the rapalogues, have not been successful as monotherapy for recurrent GBM, either, and may lead to increase activation via loss of negative feedback and reactivation of the pathway via AKT [16].

Since monotherapy with either EGFR inhibitors or rapalogues does not provide tumor control, a rational approach to overcome the resistance to EGFR inhibitors in tumors with PTEN loss might include combining treatments with both an EGFR inhibitor and an inhibitor of the PI3K pathway for synergistic therapeutic success [17]. An early pilot study found that the combination of a receptor tyrosine kinase inhibitor such as gefitinib or erlotinib with the mTOR inhibitor sirolimus can lead to tumor response [18]. However, a subsequent phase II study of erlotinib and sirolimus that utilized standard, single-agent doses of each agent, was conducted in 32 patients with recurrent GBM, but no response was seen with this combination [19].

In previous monotherapy trials, the doses of erlotinib have ranged from 150 to 200 mg for patients not on enzyme-inducing seizure medications [15, 20]. Our phase I trial of the mTOR inhibitor rapamycin was able to reach a daily dose of 10 mg [16]. Previous studies of erlotinib and sirolimus, however, used lower dosages of these drugs, usually 150 mg for erlotinib and 4–5 mg daily of erlotinib without seeing any significant toxicities and infrequent grade III or greater events [18, 19]. Therefore, we conducted a phase I trial of erlotinib in combination with sirolimus to determine the feasibility of escalating the doses in this combination to the maximum dosages seen in monotherapy. This manuscript reports the results and maximum tolerated dose (MTD) of this combination in patients with recurrent malignant glioma.

## Patients and methods

### Patient population

Eligible patients were ≥18 years of age with recurrent malignant gliomas (pathologically confirmed WHO grade III or IV). Patients must have unequivocal radiographic evidence of disease recurrence by Macdonald criteria and evaluable or measureable disease on either magnetic resonance imaging (MRI) or computed tomography (CT). There were no restrictions on the number of previous recurrences and treatments, but patients must have failed treatment with radiation therapy. Eligibility criteria also included KPS ≥60 and adequate hematologic and organ function. Patients were excluded if they received previous treatments with EGFR-inhibitors or mTOR inhibitors, were receiving enzyme-inducing antiepileptic drugs, were diagnosed with psychiatric disorders, or were pregnant or breastfeeding. The protocol and informed consent were approved by the University of California at Los Angeles (UCLA) Institutional Review Board. All patients reviewed, signed, and provided written informed consent before enrollment.

### Study design

This study was a phase I dose-escalation trial to establish the MTD of the combination of erlotinib and sirolimus. The study was also designed to define the safety profile of this combination.

### Dosing and escalation

Erlotinib (Tarceva^®^) was supplied by Genentech, Inc.; South San Francisco, CA; and was given once a day in combinations of 25, 100, and 150 mg tablets. Patients were instructed to take these tablets in the morning with up to 200 ml of water 1 h before and 2 h after food. Erlotinib was started on Cycle one day 1 of the study.

Sirolimus (rapamycin: Rapamune^®^: Wyeth-Ayerst, PA, USA) is commercially available in 1 mg tablets. In order to establish pharmacokinetic data and toxicities related to erlotinib alone, sirolimus was not started until day 8 of cycle one with a loading dose, usually three times the daily maintenance dose. Starting day 9, sirolimus was given at a once daily maintenance dosage. This study drug also needed to be taken on an empty stomach with avoidance of grapefruit juice.

Dosages of erlotinib and sirolimus for each dose level were given according to the Dose escalation scheme (Table 2). Dose escalation was performed in cohorts of three patients beginning at a starting dose of erlotinib of 150 mg per day and maintenance sirolimus of 5 mg per day (loading dose of 15 mg) (Dose Level 1). If no dose-limiting toxicity (DLT) occurred in that cohort, a subsequent cohort of three additional patients opened at the next dose level. If one patient experienced a DLT, three more patients were added to that dose cohort. The MTD was defined as the dose at which no more than one in six patients experienced a DLT and at which the next higher dose exceeded that limit, or the maximum planned dose level. Patients had to complete at least 21 days of the combination of treatments or experienced DLT within the first cycle of treatment to be evaluable for safety and DLT. A cycle of treatment was defined as 28 days starting on the first day of erlotinib.

In the first version of our protocol, the maximum dose level allowed for titration (dose level 3) was erlotinib 200 mg and sirolimus daily dose of 7.5 mg, based on single agent MTDs for these two drugs. Dose level 3 would have been achieved by dose escalation for erlotinib first (level 2), followed by dose escalation for sirolimus (level 3) if no DLTs at Dose Level 2. However, we later amended the protocol to try dose escalation of sirolimus only (Dose Level 2b) when dose level 2 had more than two DLTs.

### Patient evaluation

Pretreatment evaluation included a medical history and physical examination. Baseline tumor measurements by MRI or CT and baseline laboratory studies for hematology and chemistry were obtained within 14 days before study entry. Baseline EKG, Chest X-ray, and fasting lipid levels were also obtained during this time. Hematology, chemistry, and cholesterol panels were repeated every 4 weeks along with a complete physical and neurological examination for the first two cycles, then subsequently every other cycle. MRI/CT was performed every 4 weeks for the first two cycles then every 8 weeks for subsequent cycles. Patients with stable or responding disease continued the combination of erlotinib and sirolimus at the same dose level unless a DLT occurred in which case they received a reduced dose at the next cycle.

DLT was evaluated according to the National cancer institute common toxicity criteria ver. 3. DLT was defined as any grade IV hematologic toxicity, any nonhematologic grade III toxicity (except for diarrhea or rash at grade III that were not maximally medically treated), and failure to recover from toxicities within 3 weeks from the last dose of study drug. The toxicities of rash and diarrhea were considered DLTs only if they remained at grade III or greater despite maximal medical treatments and required more than 21 days of dose interruption or dose reduction. Patients were eligible for DLT determination only if they have taken study drugs for more than 21 days or had a grade III or higher toxicity attributable to study drugs.

Sirolimus trough levels were obtained 3 weeks after the start of sirolimus dosing, on day 28 of cycle one, and at the end of subsequent cycles. The levels were evaluated by the UCLA clinical laboratories. We were unable to evaluate serum levels for erlotinib.

Tumor progression was defined as a new enhancing lesion representing tumor greater than 1 cm^2^ in size, tumor growth of >25 % of the enhancing tumor at stable steroid dosage, failure to return for evaluation due to death, or deteriorating neurological condition.

### Statistical considerations

The primary end points for this phase I study of erlotinib and sirolimus were to define DLT and determine the MTD for dosing in a phase II trial. The dose for patients was escalated as described, and DLT, MTD, and safety were evaluated. A classical ″3 + 3″ study design was used to determine the MTD, where the MTD is defined as the dose level at which no more than 1/6 patients experienced a DLT, and two or more patients experienced a DLT at the next higher dose level. The DLT-evaluation period was the first 28 days of treatments. A patient was replaced if the patient was not evaluable for toxicity for at least 28 days of treatments and did not experience a DLT within the first cycle of treatment.

This phase I study also had an exploratory secondary endpoint of efficacy with evaluation for median PFS and response rate.

## Results

### Patient characteristics

A total of 19 patients were enrolled between December 2007 and June 2010 (Table 1). All patients failed treatments with both radiotherapy and temozolomide chemotherapy. Most of the patients (14 / 19) had GBM and were treated in the 1st or 2nd recurrence. Patients were enrolled in three different dose levels in cohorts of 3, with replacement if a patient did not meet criteria for safety evaluations for DLT as defined above (Table 2).

**Table 1:** Patient Characteristics

Number	19
Gender	
Male	14
Female	5
Age, median (range)	49 (31–71)
Pathology	
GBM	14
AA	2
AMG	2
AO	1
Recurrence	
1st	5
2nd	8
3rd	5
4th	1
KPS, median (range)	80 (70–90)

*GBM* Glioblastoma, *AA* Anaplastic astrocytoma, *AMG* Anaplastic Mixed Gliomas, *AO* Anaplastic Oligodendroglioma, *KPS* Karnofsky Perfomance Scale

**Table 2:** Dose Levels And Enrollment

Dose Level	Erlotinib dose (mg)	Sirolimus dose (mg) (loading/ maintenance)	Number enrolled	Number DLT	Number replaced	Number stopped for DP
−1	150	9/3	0			
1	150	15/5	11	1	5	6
2	200	15/5	3	2	0	3
2b	150	30/10	5	2	1	4

### Toxicity

In the DLT evaluation period, there was one grade III neutropenia in the first cohort. At this level, three subjects subsequently withdrew consents to continue study prior to completing the DLT evaluation period. One subject was unwilling to continue follow-up due to distance, and two other subjects had grade II but intolerable side effects did not want to continue with the trial. The remaining two subjects did not experience any DLT. Upon dose escalation to level 2, two subjects developed DLTs with prolonged grade III mucositis (1) and grade III rash (1), despite maximal medical managements. At this point, the protocol was amended to titrate up sirolimus instead of erlotinib (dose 2b). However, two more subjects had DLTs at this level with one grade III hypophosphatemia and one Serious Adverse Events with hospitalization for altered mental status, without tumor progression, in the DLT evaluation period. At this level, one subject also withdrew consent prior to completing the first cycle. As a result, dose level 1 was expanded to another three patients (with two subjects withdrawing consent before evaluable for DLTs) and had no further DLTs. Therefore, the MTD was determined to be dose level 1 (Table 2).

The most frequent toxicities attributable to erlotinib and/or sirolimus, including those occurring outside of the DLT period and in more than 20 % of subjects, are listed in Table 3. In summary, most of the toxicities were expected side effects. Rash was the most frequent toxicity which occurred in almost all (17 / 19) patients, usually within 1 week of treatment with erlotinib. Most of the rash side effects were grade I and II, although there were four events with grade III rash. Rash was easily managed with topical corticosteroids and oral minocycline in most cases and usually dissipated within a few days of initiating treatments.

**Table 3:** Toxicities

Adverse Event Description	Grade I	Grade II	Grade III	Grade IV	Total	Number of subjects
Diarrhea	12	3	5	0	19	14
Nausea	3	4	1	0	8	6
Rash	16	12	4*^#^	0	31	17
Dehydration	0	5	4	0	0	8
Fatigue	2	10	3	0	13	11
Stomatitis/mucositis	5	6	3*^#^	0	13	10
Anorexia	4	1	2	0	6	8
Hypercholesterolemia	1	3	2	0	5	6
Hypophosphatemia	0	1	1*	0	2	1
Dry skin	3	1	2	0	6	5
Skin breakdown/wound	1	3	2	0	6	4
Leukopenia	1	3	4	0	8	3
Neutropenia	1	2	1*	0	4	3
Thrombocytopenia	12	3	1	0	16	11
Headache	1	3	1	0	5	4
Altered mental status	1	1	1*	0	3	3
Photosensitivity	3	0	1	0	2	3
Confusion	1	0	1	0	2	2
Deep Venous Thrombosis	0	0	1	0	1	1
Pulmonary Embolism	0	0	0	1	1	1
CNS bleed	0	0	1	0	1	1

*DLTs

^#^only one event leading to DLT despite maximal medical management

Mucositis was another frequent adverse event after about 1–2 weeks on combination therapy, mostly at grade I–II, but one subject did have grade III mucositis despite maximal medical interventions leading to a DLT. Other frequent grade III non-hematological toxicities, occurring outside of the DLT-evaluation period, included diarrhea, fatigue, dry skin, and wound breakdown. Rare grade III events were seen with anorexia, headache, nausea, and deep venous thrombosis. One subject did experience grade IV toxicity with pulmonary embolism, and one patient had an asymptomatic intraparenchymal hemorrhage incidentally detected on imaging.

The most frequent hematological toxicity was thrombocytopenia, with one grade III thrombocytopenia. There were several patients with grade III leukopenia and one with grade III neutropenia. None of the subjects developed severe hyperglycemia, and one subject developed grade III hypercholesterolemia.

### Pharmacokinetic data

Sirolimus trough levels for patients evaluable for DLT are listed in Table 4. Overall, the dose levels were variable in the different cohorts. Although the highest drug level was seen in Cohort 2b, the serum level does not correlate with the degree of toxicity (data not shown).

**Table 4:** Sirolimus Levels

Dose level	Erlotinib dose (mg)	Sirolimus dose (mg) (loading/ maintenance)	Sirolimus mean levels (mg/ml)	Sirolimus levels range (mg/ml)
1	150	15/5	13.0	9.4–21.6
2	200	15/5	10.0	7.2–12.8
2b	150	30/10	22.9	13.6–35.2

Sirolimus mean levels were obtained from the PK draw in the first cycle

### Survival data

Although survival was not the primary objective of this phase I study, we did follow patients for median PFS. None of the patients remained on study beyond 6 months. The median PFS was 28 days on this treatment for all dose levels. The best tumor response was stable disease, and no objective response was seen with the combination of treatment.

## Discussion

In conclusion, this phase I study of the combination of sirolimus and erlotinib found the MTD for this combination to be erlotinib of 150 mg per day and sirolimus of 5 mg per day. These dosages are lower than dose levels reached by prior studies of single agent erlotinib or sirolimus [16, 20] and definitely not an improvement in the previous phase II trial of this combination [19]. Since we were unable to achieve higher dosages than previous unsuccessful clinical trials of erlotinib and/or sirolimus, therefore, we terminated the trial and did not proceed with the phase II trial of this combination.

Given that malignant gliomas can have multiple aberrant molecular targets, combination targeted therapies likely have more efficacy than single agent therapies [17]. Single agent molecular targeted trials in recurrent glioblastoma have shown little improvement in survival [21]. However, multiple combination therapies, including those targeting EGFR pathway and/or PI3K pathway still did not show an improvement in survival over single agents [14] Furthermore, from the experience of this phase I trial, combination therapies can also be limited by increased toxicities due to overlapping target inhibitions at higher than minimal dosages.

Our subjects experienced toxicities well-known in the side effect profiles of both drugs, including rash and diarrhea for erlotinib, and mucositis for sirolimus, increasing the frequency of DLTs. The combination also seems to have higher frequencies of fatigue, altered mental status, and mucositis than expected with single agents. Many subjects also withdrew consents before even developing any DLTs, suggesting subjective intolerability to this combination. Other combinatorial studies for treatments of GBM have also resulted in increased toxicities when combining agents without improved efficacy, possibly from pharmacokinetic interactions between the two drugs [22, 23]. Serum levels for sirolimus did not seem to correlate with the degree of toxicity. Unfortunately, our current study could not determine the potential pharmacokinetic interactions between the two therapies since we were unable to evaluate drug levels for erlotinib. However, the sirolimus levels at each dose cohort were similar to the range seen in our previous phase I study of sirolimus alone [16], suggesting that the addition of erlotinib did not significantly alter the pharmacokinetic of sirolimus.

Future clinical studies on molecular treatments should consider other strategies to achieve better target inhibition without additional toxicity. Choice of agents for combination treatments should be based on both logical molecular targets and complimentary side effect profiles. Another option includes changing clinical trial design to allow intrasubject dose escalation, so an individual subject may reach higher dosing without DLTs, or using a sequential treatment paradigm [21]. Other options include using alternative drug schedules, such as pulsatile dosing to achieve better Central Nervous System (CNS) drug level [24, 25] with the potential to limit exposure to prolonged toxicities, as seen with the currently enrolling trial using pulsatile dosing of erlotinib for recurrent GBM [26].
